# Microglial Responses after Ischemic Stroke and Intracerebral Hemorrhage

**DOI:** 10.1155/2013/746068

**Published:** 2013-10-10

**Authors:** Roslyn A. Taylor, Lauren H. Sansing

**Affiliations:** ^1^Department of Immunology, University of Connecticut Health Center, Farmington, CT 06032, USA; ^2^Department of Neuroscience, University of Connecticut Health Center, Farmington, CT 06032, USA; ^3^Department of Neurology, University of Connecticut Health Center and Hartford Hospital, Hartford, CT 06102, USA; ^4^Department of Neurosurgery, Hartford Hospital, Hartford, CT 06102, USA

## Abstract

Stroke is a leading cause of death worldwide. Ischemic stroke is caused by blockage of blood vessels in the brain leading to tissue death, while intracerebral hemorrhage (ICH) occurs when a blood vessel ruptures, exposing the brain to blood components. Both are associated with glial toxicity and neuroinflammation. Microglia, as the resident immune cells of the central nervous system (CNS), continually sample the environment for signs of injury and infection. Under homeostatic conditions, they have a ramified morphology and phagocytose debris. After stroke, microglia become activated, obtain an amoeboid morphology, and release inflammatory cytokines (the M1 phenotype). However, microglia can also be alternatively activated, performing crucial roles in limiting inflammation and phagocytosing tissue debris (the M2 phenotype). In rodent models, microglial activation occurs very early after stroke and ICH; however, their specific roles in injury and repair remain unclear. This review summarizes the literature on microglial responses after ischemic stroke and ICH, highlighting the mediators of microglial activation and potential therapeutic targets for each condition.

## 1. Introduction

Microglia are the resident immune cells of the central nervous system (CNS). The majority of our understanding about microglial responses to injury comes from rodent models. In mice, microglia arise from hematopoietic progenitors in the yolk sac at E8 in development [1]. Under normal physiological conditions, microglia self-renew and locally expand to maintain their numbers. However, blood-derived cells have been shown to regenerate microglial populations under scientific manipulations [2]. Microglia survey the CNS and phagocytose debris under homeostatic conditions as well as in injury and disease [3]. Microglia express Toll-like receptors (TLR) and NOD-like receptors (NLR), allowing them to detect bacterial pathogens and molecular signatures of injury, leading to the transcription of proinflammatory cytokine genes. Local [4] and systemic [5] infections, neurodegenerative conditions [6], and sterile injury [7] have been reported to activate microglia.

**Figure 1:**
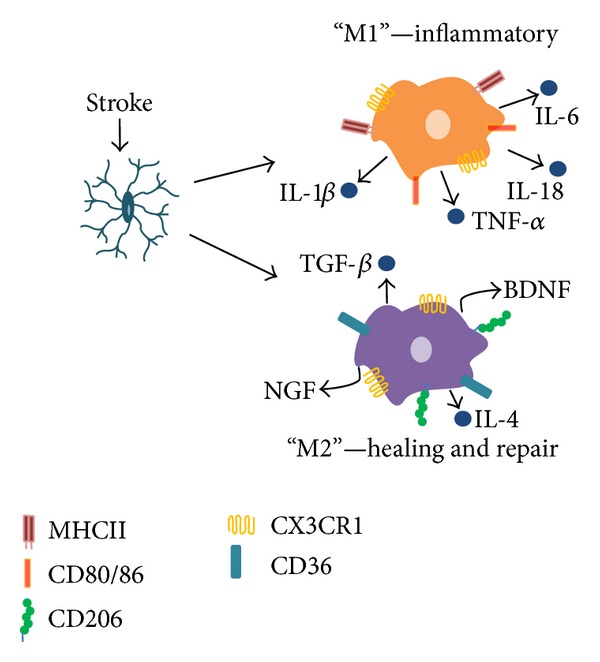
Microglia polarization is characterized by distinct phenotypes.

Once activated, microglia retract their ramifications and obtain an amoeboid morphology, becoming indistinguishable from activated macrophages. Both cell types derive from primitive myeloid cells, causing them to express many of the same markers (CD11b, F4/80, Iba-1) [1, 8], Due to this, studying microglial activity by immunohistochemistry has been difficult, causing researchers to identify activated phagocytes as microglia/macrophages. However, with the use of flow cytometry, populations of microglia and macrophages can be separated and studied individually due to their differences in CD45 expression [9]. Like macrophages, microglia can have either an M1, classically activated phenotype, or an M2, alternatively activated phenotype (Figure 1). M1 microglia are considered to be proinflammatory and secrete TNF-*α*, iNOS, and CCL2. They express CD80, CD86, and MHCII on their cell surface, possessing the capability to present antigens to T cells [10]. They express IL-23, giving implications that microglia T-cell crosstalk may occur [11]. Microglia also produce IL-1*β* and IL-18 through activation of the inflammasome [11]. M2 microglia are interpreted to be healing cells that are involved with neuroprotection and repair after injury with arginase activity and upregulation of neurotrophic factors [10, 12]. Because of their polarity, microglia have the potential to be both injurious and neuroprotective in CNS disease and injury. Here, we will review the role of microglia in neuroinflammation and acute injury after ischemic and hemorrhagic stroke.

## 2. Cerebral Ischemia

Stroke is the 4th leading cause of death in the United States, affecting 7 million people [13–15]. Ischemic stroke constitutes 87% of all strokes and is caused by the occlusion of a blood vessel due to either embolism or thrombus. As a result, brain tissue is deprived of blood glucose and oxygen [13]. This leads to neuronal death, release of reactive oxygen species, activation of complement, and upregulation of adhesion molecules on endothelial cells. Dying cells release danger signals into the environment, including HMGB1 and ATP, activating microglia [16]. This cascade of events leads to glial toxicity and infiltration of peripheral leukocytes [14, 16]. The treatment for ischemic stroke is tissue plasminogen activator (tPA) which degrades the clot in the blood vessel in order to restore perfusion to the brain. However, tPA can only be administered to patients within a 3-to-4.5-hour window after the onset of stroke, and the majority of stroke patients are left with some infarction despite treatment [17, 18]. With reperfusion of the brain or collateral circulation, peripheral leukocytes can infiltrate the brain to the area of injury and secrete proinflammatory cytokines, thus leading to secondary injury [14].

Microglial activation and proinflammatory cytokine production have been well characterized in rodent models of ischemic stroke. Early studies using flow cytometry show an increase in microglial populations in the ipsilateral hemisphere, whereas the contralateral hemisphere remains at basal levels [9]. As resident immune cells in the CNS, microglia are known to both phagocytose debris and secrete proinflammatory cytokines under ischemic conditions, contributing to tissue damage [19]. Under ischemic conditions, microglia can become destructive and phagocytose tissues as well. However, microglia have been reported to also secrete anti-inflammatory cytokines such as IL-10 and TGF-*β* [20–22], which act to quell inflammation. In studies where microglia have been ablated, mice had larger infarctions and a doubling of apoptotic neurons after ischemia, indicating the importance of microglial activity after ischemic stroke [23]. Thus, while microglia can be destructive in repair and recovery, their presence is needed to alleviate injury. The balance of these processes may depend on the location of the microglia, the degree of ischemia, and the timing after injury.

### 2.1. Microglial Activation in the Ischemic Core

The location of microglia in the ischemic brain changes their activation and cell fate. In the ischemic core, where blood flow is reduced to near zero, cell death is nearly universal by 24 hours [24]. In a 90-minute transient ischemia model, degenerating Iba1+ microglia are apparent 3.5–12 hours after reperfusion. Over 24–48 hours, round Iba1+ED1+ cells appear throughout the core [25]. Immediately after 60 minutes of focal ischemia without reperfusion, microglia/macrophages in the striatum (ischemic core) significantly increased the number of their processes. Twenty-four hours later, the microglia/macrophages in the ischemic core showed a reduction in numbers of processes, had significantly shorter processes, and increased CD11b expression indicating activation and the formation of an amoeboid morphology [24]. Twenty-four hours after permanent MCAO, few double positive CD11b+CD68^+^ (a marker for phagocytosis) cells were found in the ischemic core; however, by day 7, CD68 expression increased. At 24 hours, M2 markers Ym1 and CD206 were exclusively found in the ischemic core, suggesting that the microglia/macrophages in the ischemic core promote tissue repair [26]. These findings were corroborated by another study examining the location of M1 versus M2 microglia/macrophages, which found M2 microglia/macrophages infiltrating the ischemic core at 24 hours, peaking at 5 days, and declining in the striatum by 14 days [27]. In contrast, using the inflammatory marker CD16/32 (Fc*γ* receptors), they found M1 cells increasing in number in the striatum over time and outnumbering the M2 cells during the second week. However, nonvital (measurement of vitality undefined) cellular debris found in the ischemic core at 72 hours after photothrombosis in rats exhibited CD11b staining, indicating that large numbers of microglia/macrophages in the ischemic core are destined to die [28]. Likewise, at 7 days after TiO_2_ sphere-medicated ischemia, CD11b+ cells in the ischemic core “showed signs of disintegration” [29]. These studies show that the microglia in the infarct core are initially injured as a result of ischemia. M2 microglia/macrophages then enter the area during the first week before declining in numbers, while M1 microglia/macrophages increase in numbers over the first 2 weeks.

### 2.2. Microglial Activation in the Peri-Infarct Zone

Microglia in the peri-infarct zone have different patterns of microglial activation than those found in the ischemic core. After 90 minutes of transient ischemia, activated Iba1+ED1− cells increased in number from 3.5 to 7 days after reperfusion but were decreased by day 14 [25]. In another study, eight and twenty-four hours after transient MCAO in mice, microglia/macrophages in the border zone had shorter processes with fewer endpoints indicating activation. CD11b and F4/80 expression were the greatest on those microglia/macrophages closest to the infarct border, localizing their activation to the site of injury [24, 30]. Ym1 and CD206+ cells were not found in the peri-infarct zone at either 24 hours or 7 days in one study [26], while another found CD206+ cells in the cortex at the border zone of ischemia peaking at day 5 after ischemia before being outnumbered by M1 cells [27]. In both a photothrombosis model in rats and permanent MCAO in mice, the majority of the microglia/macrophages expressing CD68 were localized to the border zone and peri-infarct region [26, 28]. Microglia in the peri-infarct zone proliferated 48 and 72 hours after middle cerebral artery occlusion (MCAO); however, the amount of proliferation is reduced after 60-minute compared to 30-minute ischemia [31]. This work used CFSE and BrdU injection to distinguish between microglia and blood-derived macrophages histologically and determined that microglial proliferation, not macrophage recruitment, led to the increase in peri-infarct myeloid cells. By 72 hours after stroke, microglia/macrophages at the infarct border and peri-infarct region coexpress CD68 and MHCII, a marker of antigen presentation. By 7 days, the infarct was demarcated by a GFAP+ glial scar and double positive CD68^+^, MHCII^+^ phagocytic cells [32]. Together these studies indicate that the peri-infarct region is dominated by proinflammatory, proliferating, and activated microglia that increase in number over the first week after ischemia. These studies reveal that the dynamics of microglial phenotypes change over time and that the location of the microglia (core versus penumbra) is critical in determining that phenotype. However, these studies have been limited by an inability to differentiate between infiltrating macrophages and microglia, which both likely contributed to the measured phenotypes.

Bone marrow chimeras have been helpful in distinguishing the activated microglia from macrophages after ischemia. In this experimental paradigm, C57Bl/6 mice were irradiated and the hematopoietic system reconstituted with bone marrow from mouse expressing GFP in leukocytes. Resident microglia, due to their radioresistant nature, would remain of host origin (GFP^−^). Twenty-four hours after MCAO, activated, GFP^−^ microglia were seen in the infarct region [33]. At 48 hours after stroke, GFP-Iba-1^+^ microglia ingested neuronal debris [34]. By 4 days after infarct, numerous GFP^+^, F4/80^+^ phagocytic cells were seen in the infarct area. These studies indicate that the activated, phagocytic F4/80^+^ cells identified in the early time points after ischemia are likely microglia [33, 34]. While these studies help elucidate the role of microglia from monocytes after ischemia, it is important to note that radiation may alter the blood brain barrier. In a West Nile Virus infection model, bone marrow chimeras reconstituted with bone marrow from a GFP^+^ mouse showed resting, ramified microglial-like cells which were GFP^+^, suggesting that blood-derived monocytes traffic into the brain after radiation and obtain a microglial morphology [35]. In order to properly study the role of microglia after stroke using bone marrow chimeras, heads need to be protected from radiation with a lead shield. Alternatively, a parabiosis model may be employed, although this precludes the ability to perform functional outcome testing.

### 2.3. Microglial Cytokine Production after Ischemic Stroke

Microglial cytokine production can be seen as early as 1 hour after stroke. Microglia have been reported to be the primary source of IL-1*β* in a biphasic time course peaking at 1 hour and 24 hours. IL-1*β* deficient mice have significantly smaller infarct volume 24 hours after permanent MCAO [36]. Microglia have also been reported to be the main source of IL-6, TGF-*β*, and IL-10 after ischemia [37]. RhIL-6 treatment 30 minutes prior to and 15 minutes after permanent MCAO notably reduced infarct size [38]. Within 4 hours after stroke, TNF-*α* production can be seen within and surrounding the infarct. Microglial/macrophage production of TNF-*α* can be measured 6, 12, and 24 hours after ischemic stroke [19]. However, the role of TNF-*α* production in ischemic stroke still remains controversial. While some studies found that TNF-*α* antagonism resulted in improved outcomes [39, 40], other studies found that TNF-*α* is important in hippocampal and striatal neurogenesis [40–42].

### 2.4. Mediators of Microglial Activation

The identification of specific mediators of microglial activation may provide a therapeutic target to alleviate microglial-mediated injury (Table 1). Recent work has identified galectin-3 as an important survival factor in microglial survival, proliferation, and migration [23, 43] after ischemic injury. *In vivo*, microglia/macrophages upregulate galectin-3 72 hours after MCAO. In culture, WT microglia upregulate Iba-1, TLR2, CD68, and galectin-3. However, galectin-3^−/−^ microglia were only capable of upregulating Iba-1, indicating that galectin-3 plays a role in the upregulation of TLR2 and CD68 [23]. The absence of galectin-3 led to larger areas of infarction and enhanced neuronal apoptosis after MCAO [23]. The role of Notch signaling has also been studied in microglial activation. Notch signaling occurs during inflammation and had been correlated to worse outcome after stroke. Transgenic mice with an antisense Notch have less CD11 staining; in the presence of LPS, microglia had less CD11b expression. In culture exposed to oxygen glucose deprivation (OGD), activated WT microglia upregulate Notch expression. Antisense Notch mice, when injected with LPS or subjected to MCAO, produced less TNF-*α* and IL-1*β* and also had attenuated NF*κ*B p65 activity, indicating that Notch signaling may play a role in microglia toxicity after ischemia [44]. These studies indicate that galectin-3 provides a mechanism for reducing cell death and infarction after cerebral ischemia, while Notch signaling leads to enhanced inflammation and increased microglial neurotoxicity.

Secreted protein acidic rich in cysteine (SPARC) is a matricellular protein that regulates growth factors and the assembly of the extracellular matrix. SPARC has been shown to play a role in microgliosis after ischemia. Under homeostatic conditions, mature microglia express SPARC. In a focal photothrombotic cortical ischemic stroke model, microglia downregulate SPARC expression after injury. SPARC-null mice show increased processes length and branching at steady state in white matter. Microglial expansion is significantly increased in grey matter and reduced in white matter in the SPARC-null mice, indicating that SPARC plays a differential role in microglial expansion depending on the location in brain. By immunohistochemistry, SPARC-null mice had an increase in Gal-3 expression, indicating higher levels of microgliosis, which correlated to better functional outcome after cortical ischemia [45]. The evidence points to a role for SPARC in microglial toxicity and poor outcome after cerebral ischemia.

High motility group protein B1 (HMGB1) may act as a cytokine to activate microglia after ischemia [46]. HMGB1 increases in the blood and cerebral spinal fluid in rats after ischemia [46] and induces postischemia neurodegeneration [47]. When HMGB1 was reduced using a shRNA transgene injected into the striatum, the number of microglia in the infarct was reduced. Those in the infarct maintained a ramified morphology and had less p38 MAPK activity and TNF-*α*, IL-1*β*, COX2, and iNOS expression. *In vitro*, HMGB1 was released after incubation of microglial cultures with NMDA-treated cortical cells. Cultured microglia remained quiescent in this model when an HMGB1 inhibitor was introduced into the media with the cortical cells, identifying HMGB1 as the mediator of inflammation [46]. HMGB1 induces activation via the RAGE receptor on both microglia and blood-derived macrophages after ischemia [48]. Taken together, these studies indicate that HMGB1 may be a potential therapeutic target for modulating microglial activation and mediated injury.

The chemokine receptor CX3CR1 is expressed at high levels on murine microglia under homeostatic conditions. Its ligand, CX3CL1 (fractalkine), is produced by neurons and can be either secreted or membrane bound. When it is membrane-bound, neuronal CX3CL1 binds to microglial CX3CR1 and maintains microglia in a quiescent state [64, 65]. Cleaved CX3CL1 acts as a chemokine to induce microglial chemotaxis [49]. The CX3CL1-CX3CR1 interaction has been shown to regulate microglial toxicity in models of Parkinson's, ALS, and LPS activation—when the receptor is nonfunctional, mice have worse functional outcome [65]. However, this neuroprotective role for microglial CX3CR1 signaling may not be preserved after ischemia. CX3CL1 expression is upregulated within 48 hours after MCAO and decreases by 7 days, while CX3CR1 expression was the highest on the microglia within the infarcted tissue at 7 days. These results suggest that this pathway may play a role in microglia/macrophage cell recruitment into the infarcted region [50]. However, mice genetically deficient for either CX3CR1 or CX3CL1 have smaller infarct volumes after MCAO [51–54]. This was accompanied by fewer blood-derived leukocytes infiltrating the brain at 72 hours and improved functional outcomes [53]. After 30 minutes of ischemia, CX3CR1^−/−^ microglia remained in a ramified state, whereas WT microglia/macrophages had amoeboid morphology at 24 hours. WT microglia/macrophages had a proinflammatory profile with elevated iNOS and CD68 expression, whereas CX3CR1^−/−^ microglia/macrophages had low expression of iNOS and CD68 but elevated Ym1 implicating a healing phenotype [52]. The addition of exogenous CX3CL1 intracerebroventricularly prior to MCAO in WT resulted in smaller infarct [54]. These studies indicate that global deficiency of this signaling pathway in both microglia and peripheral leukocytes is protective after ischemia. Through the use of bone marrow chimeras, the specific role of microglial CX3CR1 in infarct volume, functional outcome, and neuroinflammation can be better understood. It is possible that the differences observed in these studies were in part due to CX3CR1 deficiency on the peripheral monocytes. It has recently been reported that human bone marrow stromal cells transplanted into rats after MCAO used the fractalkine-CX3CR1 pathway to migrate to area of infarct [66]. In addition, in a clinical study, it was found that patients with lower concentrations of plasma CX3CL1 had worse outcome 6 months after stroke [67]. The apparently conflicting roles of CX3CL1-CX3CR1 signaling may be due to alterations in signaling pathways in genetically altered mice, opposing roles of this pathway on microglia and CX3CR1+ macrophages or other factors.

### 2.5. Treatments Targeting Microglial Activation after Cerebral Ischemia

While tPA is available to aid in reperfusion for selecting patients with ischemic strokes, there is a necessity for treatments to reduce injury and aid in repair after stroke. Microglial activation is one potential target. Several studies have investigated the role of minocycline on inhibiting or altering microglial activation. In a mouse model of ALS, minocycline attenuated microglial activation and reduced the expression of M1, but not M2, microglia/macrophage markers suggesting that minocycline inhibits the proinflammatory microglia/macrophages [68]. In mice, minocycline administered two hours after transient MCAO reduced infarct volume by 25% [69]. Rats which received continual minocycline treatment for 4 weeks after ischemia had reduced microglial activation by microscopy, which correlated with increased neurogenesis and better functional outcome [70]. Transplantation of bone marrow mononuclear cells (BMMC) is also being investigated as a possible treatment for ischemic stroke [71–73]. *In vitro*, BMMCs reduced neuronal death due to LPS and hypoxia activated mixed culture of microglia and peritoneal macrophages. Microglial cultures in the presence of BMMCs had higher levels of IL-10, VEGF, IGF1, and SDF-1a [74]. Recent studies have investigated whether the addition of minocycline can improve functional outcome and neuroprotection after BMMC transfer after ischemia *in vivo*. Rats that received minocycline and BMMC treatment had reduced CD68^+^ cells by immunohistochemistry and better functional outcome [75, 76]. These studies suggest that microglia contribute to neuroinflammation after ischemia and that BMMC therapy and minocycline have additive effects in reducing poststroke microglial activation. However, the dosing of minocycline is crucial for benefit; high doses induced toxicity in both neurons and astrocytes [77]. Minocycline is a tetracycline antibiotic that is not specific for microglia [77], and off-target effects may contribute to dose-limiting toxicities.

While many studies have focused on understanding the mechanism by which microglia cause secondary injury after stroke, other studies have shown that microglia are essential for prevention of neuronal apoptosis [23]. Microglia are needed for recovery and repair after ischemia. Rats transplanted with a human microglial cell line 48 hours after MCAO had better functional outcome 7, 10, and 14 days after stroke. The improved functional outcome correlated with fewer apoptotic cells, fewer CD68 phagocytic cells, and less GFAP glial scar in the ipsilateral hemisphere. Notably, rats that received human microglia had less endogenous microglial activation and upregulation of IL-4, IL-5, and neurotrophic factors, thus decreasing their neurotoxicity. Human microglial cells reduced ischemic injury and promoted repair in rats [78]. Therefore, microglia have potential for augmenting repair after ischemia and studies on the modulation of their phenotype towards healing processes may identify new therapeutic targets for stroke.

## 3. Intracerebral Hemorrhage

Research on the mechanisms of injury and repair after ICH has been more limited than after cerebral ischemia. ICH occurs when a blood vessel in the brain parenchyma ruptures, most commonly due to hypertension [79]. ICH has a high mortality rate: 30–50% of patients die within the first 30 days [79]. Of those who survive, only 20% regain independence within six months [80]. Despite recent advances in ICH research, no specific treatment for ICH currently exists [81]. The introduction of blood components, including thrombin, heme, and leukocytes and platelets, into the brain creates the basis for a secondary injury due to microglial activation and neuroinflammation resulting in the recruitment of leukocytes into a normally immune privileged site [82, 83].

The activation of microglia likely has dual roles after ICH. While some microglial processes may be beneficial, microglia have also been shown to play a role in the secondary injury that occurs after ICH [82]. A major role of microglial cells after ICH is to phagocytose the debris and erythrocytes left in the brain after hemorrhage. They have been shown to endocytose heme and hemoglobin. These processes are mediated through scavenger receptors, such as CD36, on their cell surface [84]. They also produce proinflammatory cytokines (TNF-*α*, IL-1*β*, IL-6) [85] and chemokines (CXCL2) [86], which promotes neuroinflammation and the recruitment of blood-derived leukocytes to the brain [8, 84].

### 3.1. Time Course of Microglial Activation after Intracerebral Hemorrhage

The activation of microglia/macrophages occurs early in the timeline of neuroinflammation following ICH. Microglial/macrophage activation within the perihematomal region was seen as early as 1 hour following ICH by immunofluorescence staining in the collagenase injection model of ICH and within 4 hours using the double injection method of whole blood [62, 87]. Microglial/macrophage production of IL-1*β* in rats can be seen as early as 6 hours and can persist up to 24 hours. Interestingly, there was no IL-6 or MMP-12 staining within the activated microglial/macrophage population [88]. Twelve hours after a mouse model of ICH, microglial numbers between the ipsilateral and contralateral hemisphere did not differ, suggesting that robust proliferation or migration has not yet occurred [89]. However, by 72 hours microglia reach their peak number in the perihematomal region [8, 90], which corresponds to roughly a 40% increase in their numbers by flow cytometry [91]. A week after ICH, microglial/macrophage numbers begin to reduce; by 21 days, microglial/macrophage numbers have returned to basal levels, although some reports find that microglial/macrophage activation persists for 4 weeks [8, 90]. A time course of functional study in rats has shown a correlation between the resolution of microglial/macrophage numbers in the ipsilateral hemisphere with improvement in behavioral tests, suggesting that the presence of microglia/macrophages contributes to neurological deficit [90].

### 3.2. Mediators of Microglial Activation after ICH

Many studies have focused on the triggers of microglial activation after ICH. Blood components directly activate microglia and initiate immune responses. Thrombin, a serine protease in blood that is necessary for coagulation, causes apoptosis in neurons and astrocytes, provoking researchers to investigate whether thrombin plays a role in microglial activation after ICH [55]. In rats, direct injection of thrombin into the striatum caused neuronal apoptosis. Microglia upregulated CD11b expression and morphed from the ramified, resting state to an activated, amoeboid shape with increased p-ERK within 4 hours. By 24 hours, the activated microglia stained positive for iNOS and by 72 hours, the microglial/macrophage numbers in the ipsilateral striatum increased [56]. Thrombin-mediated activation of microglia is induced by MAPK signaling. Interestingly, MAPK inhibitors injected into the striatum prior to ICH caused a large reduction in pERK 8 hours after ICH and an increase of TUNEL positive microglia/macrophages [57]. It has also been reported that inhibitors of p38 MAPK and c-JNK inhibitors not only caused microglial/macrophage apoptosis but also greatly reduced the TNF-*α* levels. These studies suggest that the MAPK pathway in microglia/macrophages is induced by thrombin and promotes cell maintenance, allowing the production of TNF [58].

The effect of thrombin on microglial proinflammatory cytokines and matrix metalloproteases (MMPs) has also been described. In culture, microglia express thrombin receptors and when stimulated with thrombin produce IL-1*β* and TNF-*α*. Cultures treated with tuftsin fragment1-3, a microglial/macrophage inhibitory factor (MIF), had less cytokine secretion. *In vivo*, mice treated with MIF had less edema, suggesting that activated microglia are a cause of blood-brain barrier dysfunction after ICH [59]. *In vivo*, thrombin promotes the cleavage of pro-MMP9 to active MMP9. MMP9^−/−^ mice had less injury and microglial/macrophage activation than wild-type mice [60]. Another work identified neutrophils as the main source of MMP9 [92]. Interestingly, neutrophil depletion did not change microglia numbers at 3 days after ICH [93] but was found to reduce microglial/macrophage populations seven days after ICH, as well as decrease the level of CD68 on microglial/macrophage cells 3 to 14 days after ICH [92].

Products of erythrocyte lysis, including heme and iron, are also active initiators of microglial activation and neuroinflammation. Heme is converted to ferric iron, biliverdin, and carbon monoxide by heme oxygenase (HO). Iron-handling proteins, including ferritin and hemosiderin, have been found within activated microglia/macrophages after ICH [59], suggesting that microglia are responsible for iron clearance and processing. Metalloporphyrin resulted in a decrease in ferritin deposition in microglia and less neuronal loss [61]. In aged rats, treatment with deferoxamine, an iron chelator, greatly reduced the number of activated microglia/macrophages and the overall ROS production in the striatum [94]. Unconjugated bilirubin and bilirubin oxidative species are hypothesized to activate microglia *in vivo*, resulting in production of proinflammatory cytokines [95]. However, in a mouse animal model, unconjugated bilirubin infusion with the whole blood to create the ICH, there was a reduction in microglia but increase in neutrophils at 24 hours [96]. The role of unconjugated bilirubin in microglial phenotype is yet unknown. Therapies modulating iron handling by microglia may improve outcomes by reducing both iron-induced oxidative damage and inflammation.

Toll-like receptor 4 (TLR4) activation also leads to neuroinflammation after ICH. In the CNS, microglia are the most prevailing cell type expressing TLR4 [97]. TLR4-deficient mice were shown to have reduced peripheral myeloid cell infiltration and fewer microglia in the perihematomal region 3 days after ICH, along with better functional outcome [91]. Another recent study found that heme degradation products lead to production of TNF-*α*, IL-1*β*, and IL-6 via TLR4 in cultured microglia [63]; however, another work used blood transfer experiments to localize the location for TLR4 signaling to the cells in the ICH itself, rather than microglia [91]. Together, these studies indicate that TLR4 antagonism may be a potential therapeutic target for reducing microglial activation after ICH, either directly or by reducing leukocyte recruitment that then contributes to further microglial activation.

A recent study expanded upon HMGB1 acting as a proinflammatory cytokine after ICH. *In vitro,* heme stimulates cultured microglia to release HMGB1 [98]. After collagenase-induced ICH in rats, the release of HMGB1 into the cytoplasm in the brain was detected by 1 hour. The administration of ethyl pyruvate reduced the number of HGMB1^+^ cells in the ipsilateral hemisphere, improved functional outcome, and reduced edema and the number of apoptotic cells. Rats given ethyl pyruvate also had reduced numbers of activated microglia by immunohistochemistry and immunofluorescence [99]. As in ischemic stroke, targeting HMGB1 production after ICH may serve as a potential target to attenuate the immune response.

### 3.3. Treatments Targeting Microglial Activation after Intracerebral Hemorrhage

While there is currently no treatment for microglial activation after ICH, investigation into therapeutic targets is ongoing. Minocycline has been tested for neuroprotective qualities in ICH animal models as in ischemic stroke. One study administered minocycline to rats 3 hours after ICH to obtain clinical relevance. Minocycline had no effect on hemorrhage volume in either short term (5 days) or long term (28 days) survival but did reduce microglia/macrophages numbers surrounding the hematoma at 5 days [100]. In other studies, animals were given minocycline treatment 6 hours, 1 day, and 2 days after ICH. Minocycline reduced the brain water content and increased intact blood vessels 72 hours after ICH. TNF-*α* and MMP12 levels were upregulated at 24 and 72 hours after ICH, respectively. Minocycline treatment leads to a reduction in both TNF-*α* and MMP12 after ICH. However, the authors did not find colocalization of these proinflammatory factors with activated microglia/macrophages, but with neutrophils, suggesting that microglia may not be the only target of minocycline after ICH [101]. Thrombin-mediated activation of microglial cultures caused an upregulation in TNF-*α* and IL-1*β* production; minocycline treatment greatly reduced the production of both cytokines. *In vivo*, minocycline treatment reduced edema and improved functional outcome by 14 days [102]. Taken together, minocycline may serve as a promising treatment for ICH.

Peroxisome proliferator-activated receptor *γ* (PPAR*γ*) has also been investigated as a potential therapeutic for ICH. In mice, PPAR*γ* agonist treatment beginning 24 hours after ICH enhanced the phagocytosis of the hematoma and reduced IL-1*β*, TNF, MMP-9, and iNOS expression. In microglial cultures, PPAR*γ* increased CD36-mediated microglial phagocytosis of red blood cells [103, 104]. Targeting microglial function (i.e., phagocytosis) as a therapeutic for ICH may have potential for modulating the immune response and enhancing recovery. Since recovery after ICH is at least partially dependent on microglial responses, therapies that modulate these responses towards repair have promise as treatments for ICH.

## 4. Conclusions

Investigations on the role of microglia in the immune response after ischemic stroke and ICH can advance our understanding of the mechanisms of secondary injury and repair. Interestingly, the mediators of microglial activation differ between the two major types of strokes. In each condition, however, microglia can contribute to injury via the production of proinflammatory mediators and yet are crucial for remodeling and repair. Therapies that inhibit the injurious phase of microglial activation while augmenting repair would offer great promise for stroke patients. However, much of the work described above was performed on young, male rodents. The translational potential of the findings will be determined by the ability of therapies to improve outcomes across age, sex, and species. Future advances will also depend on differentiating the roles of microglia and macrophages in poststroke responses. With advances in scientific techniques, such as flow cytometry and cell sorting, the mechanisms by which microglia and macrophages contribute to neuroinflammation can be further understood, opening the possibility for new therapeutic targets.

## Figures and Tables

**Table 1:** Mediators of microglial activation after cerebral ischemia and intracerebral hemorrhage.

Mediator	Measurements used	Results	Citation
Ischemic stroke			
Galectin 3	Galectin 3^−/−^ mice subjected to 60-minute MCAO followed by reperfusion for either 24 or 72 hours	Galectin 3 reduces cell death and infarct volume	[23]
Notch	Primary cell cultures and *in vivo* models of microglial activation (LPS) and MCAO in antisense Notch mice	Notch leads to increased neuroinflammation	[44]
SPARC	Focal photothrombotic model of ischemic stroke in SPARC^−/−^ mice	SPARC^−/−^ microglia have increased processes length and branching and increased microgliosis	[45]
HMGB1	shRNA and HMGB1 inhibitor used to knock down HMGB1 in ischemic stroke model and primary microglial cultures	HMGB1 promotes neuroinflammation	[46–48]
CX3CL1	Behavioral outcomes, edema, peripheral cell infiltration, cytokine production in CX3CR1 knockout mice in 30- and 60-minute MCAO	CX3CL1-CX3CR1 signaling leads to worse functional outcome and higher neuroinflammation	[49–54]

Intracerebral hemorrhage			
Thrombin	Thrombin injection in rats and in culture caused neuronal apoptosis and increased cytokine production	Activates microglia and promotes cytokine production	[55–60]
Heme	Blood or hemin injection	Activates microglia and leads to neuroinflammation	[61–63]

SPARC: secreted protein acidic rich in cysteine; HMGB1: high mobility group box 1.
